# Maternal Calorie Restriction Induces a Transcriptional Cytoprotective Response in Embryonic Liver Partially Dependent on Nrf2

**DOI:** 10.3390/antiox11112274

**Published:** 2022-11-17

**Authors:** George I. Habeos, Fotini Filippopoulou, Evagelia E. Habeos, Electra Kalaitzopoulou, Marianna Skipitari, Polyxeni Papadea, George Lagoumintzis, Athanasios Niarchos, Christos D. Georgiou, Dionysios V. Chartoumpekis

**Affiliations:** 1 Division of Endocrinology, Department of Internal Medicine, School of Medicine, University of Patras, 26504 Patras, Greece; 2 Section of Genetics, Cell Biology and Development, Department of Biology, University of Patras, 26504 Patras, Greece; 3 Department of Pharmacy, University of Patras, 26504 Patras, Greece

**Keywords:** Keap1, Nfe2l2, reactive oxygen species, oxidative stress, gluconeogenesis, lipogenesis, integrative stress response, embryo

## Abstract

Background: Calorie restriction is known to enhance Nrf2 signaling and longevity in adult mice, partially by reducing reactive oxygen species, but calorie restriction during pregnancy leads to intrauterine growth retardation. The latter is associated with fetal reprogramming leading to increased incidence of obesity, metabolic syndrome and diabetes in adult life. Transcription factor Nrf2 is a central regulator of the antioxidant response and its crosstalk with metabolic pathways is emerging. We hypothesized that the Nrf2 pathway is induced in embryos during calorie restriction in pregnant mothers. Methods: From gestational day 10 up to day 16, 50% of the necessary mouse diet was provided to Nrf2 heterozygous pregnant females with fathers being of the same genotype. Embryos were harvested at the end of gestational day 16 and fetal liver was used for qRT-PCR and assessment of oxidative stress (OS). Results: Intrauterine calorie restriction led to upregulation of mRNA expression of antioxidant genes (*Nqo1*, *Gsta1*, *Gsta4*) and of genes related to integrated stress response (*Chac1*, *Ddit3*) in WT embryos. The expression of a key gluconeogenic (*G6pase*) and two lipogenic genes (*Acacb*, *Fasn*) was repressed in calorie-restricted embryos. In Nrf2 knockout embryos, the induction of *Nqo1* and *Gsta1* genes was abrogated while that of Gsta4 was preserved, indicating an at least partially Nrf2-dependent induction of antioxidant genes after in utero calorie restriction. Measures of OS showed no difference (superoxide radical and malondialdehyde) or a small decrease (thiobarbituric reactive substances) in calorie-restricted WT embryos. Conclusions: Calorie restriction during pregnancy elicits the transcriptional induction of cytoprotective/antioxidant genes in the fetal liver, which is at least partially Nrf2-dependent, with a physiological significance that warrants further investigation.

## 1. Introduction

In utero life is important for the development of the embryo and for its adaptation to stressors later encountered in adult life. Exposure of the embryo to stresses such as nutrient deprivation is known to cause fetal reprogramming that may contribute to the development of metabolic syndrome, type 2 diabetes and cardiovascular disease during adult life, and has been extensively reviewed [1,2,3]. A real-life example in humans is the increased incidence of obesity, diabetes and dyslipidemia in the offspring of mothers in Netherlands who were pregnant during a period of six-month famine at the end of World War II (so called the Dutch Hunger Winter of 1944–1945) [4,5,6]. Similar metabolically dysregulated phenotypes were observed in adults who were exposed in utero to the Ukraine famine of 1932–1933 [7] and to the Chinese Famine of 1959–1961 [8]. Moreover, low birth weight has been associated with increased risk of type 2 diabetes, obesity and cardiovascular disease in adult life [9,10].

Although many studies describe the resulting phenotypes of the offspring after maternal nutrient deprivation or excess, the mechanisms underlying these effects are unclear. Suggested mechanisms include permanent structural changes of organs such as pancreas, epigenetic changes such as DNA methylation, acetylation of histones and accelerated cellular aging by increased shortening of telomeres (all mechanisms reviewed in [11]). A recent study on the Dutch Hunger Winter cohort revealed an increase in DNA methylation in genes related to glucose and lipid metabolism and β-cell function [12]. A study on rats born with intrauterine growth retardation due to ligation of uterine arteries has shown distinct methylation profiles in the *PDX1* gene that plays important roles in pancreatic development and β-cell function [13]. Other researchers have shown a decreased muscle GLUT-4 (glucose transporter) expression due to histone code modifications in rats exposed to calorie restriction in utero [14]. This could contribute to the deteriorated metabolic phenotype observed in these mice.

These metabolic phenotypes of obesity and diabetes are usually associated with increased levels of reactive oxygen species (ROS) (reviewed in Ref. [15]). Long-term calorie restriction has been shown to decrease the markers of oxidative stress [16] in rat livers while long fasting/starvation could have the opposite effect [17]. However, little is known about the levels of ROS in utero during intrauterine growth retardation due to nutrients deprivation in subjects that develop metabolic disturbances in later adult life. It is true that during pregnancy a basal level of ROS is necessary as they act as signal transducers during cell differentiation, but their excess may have deleterious effects on embryonic development [18]. The embryos are relatively sensitive to OS as the expression of several antioxidant enzymes such as glutathione S-transferases and glutathione peroxidases is lower in embryos than that in adults [19]. The relevant studies for practical and ethical purposes are limited to mice. After the ninth gestational day, the placenta’s yolk sac regresses and this results in embryo exposure to higher oxygen concentrations [20] that could lead to increased levels of ROS. Nevertheless, the ROS status as well as the expression of the relevant antioxidant enzymes has not been studied in utero.

One of the central regulators of the cytoprotective/antioxidant response is the transcription factor Nuclear Erythroid Factor 2 like 2 (Nrf2) which is encoded by the *Nfe2l2* gene. Under basal conditions it is usually sequestered in the cytoplasm by Keap1, a cysteine-rich protein, that facilitates its degradation by the proteasome. Upon exposure to OS, ROS reacts with specific Keap1 cysteines and causes allosteric modifications of Keap1, rendering it unable to bind Nrf2 and lead it to degradation. Thus, Nrf2 accumulates and can enter the nucleus, where it regulates its target genes expression by binding on specific sequences called ARE (Antioxidant Response Element) present in the genes’ regulating regions [21]. Nrf2 is expressed in cells from the blastocyst stage and although expressed in all tissues, it shows high expression at embryonic day 15.5 in liver, lungs, and kidneys [22]. Deletion of Nrf2 does not cause any defects in the development of mice [22,23] with the exception of a lower placenta and embryo weight (~25%) [24]. As in adult mice, lack of Nrf2 makes embryos more susceptible to toxic insults and OS (reviewed in Ref. [25]). However, in a mouse preeclampsia model, the lack of Nrf2 did not deteriorate the phenotype but in the contrary it ameliorated it, despite the increased OS, by enhancing angiogenesis [26]. This observation reinforces the notion that the effects of Nrf2 signaling on a phenotype are not always dependent on the antioxidant/cytoprotective roles of Nrf2 but they can also be the result of Nrf2 crosstalk with other signaling pathways.

Such effects of Nrf2 beyond cytoprotection have been reported in studies on metabolism. For instance, it has been shown that Nrf2 pathway activation can repress gluconeogenesis and lipogenesis in liver and prevent fatty liver disease and ameliorate glycemic control in mouse models of obesity and insulin resistance [27,28]. Furthermore, fasting for 18 h induced Nrf2 signaling in mouse liver [29]. However, there are no studies to evaluate the effects of metabolic stressors (nutrient deprivation or excess) in utero with reference to the Nrf2-mediated (or not) stress-response. Given that Nrf2 pathway is known to be upregulated in the liver during long-term calorie restriction in adult mice [30], we sought to investigate if an in utero calorie restriction would result in similar response in embryonic liver. Our aim was to investigate if indeed Nrf2 was induced after calorie restriction and to what extent it is involved in the transcriptional metabolic adaptation of the fetal liver under this condition. Herein we used a model of maternal calorie restriction, and we show an upregulation of antioxidant/cytoprotective stress-response in fetal liver that is at least partially regulated by Nrf2. 

## 2. Materials and Methods

### 2.1. Mouse Model

All mouse experiments were performed at the University of Patras School of Medicine Animal Facility. The animal experiment protocol was approved by the relevant committee in the prefecture of Achaia in Patras, Greece and was given the number of approval 187575/630. The mice were housed at 22 °C and 50% humidity with a 12:12-h light-dark cycle with ad libitum access to water and food (standard diet 4RF21, Mucedola S.R.L., Settimo Milanese, Italy). All mice were in the C57BL/6J background. Nrf2 knockout mice (Nrf2KO) were originally developed by Prof. M. Yamamoto [23] and were obtained from RIKEN BRC (Tsukuba, Japan). Wild-type (WT) and Nrf2KO mice were generated by mating Nrf2^+/−^ (Nrf2 heterozygous) mice and the offspring were genotyped as previously described [31] using the following primers: primer 1, 5′-TGGACGGGACTATTGAAGGCTG-3′; primer 2, 5′-GCGGATTGACCGTAATGGGATAGG-3′; primer 3, 5′-GCACTCGCGAGCTCCTCCATTTCCGAGTC-3′. Thermal cycling conditions were: step 1, 95 °C for 180 s; step 2, 95 °C for 30 s; step 3, 70 °C for 30 s; step 4, 72 °C for 30 s; go to step 2 34 times; step 5, 72 °C for 120 s [32].

### 2.2. In Utero Calorie Restriction

**Figure 1:**
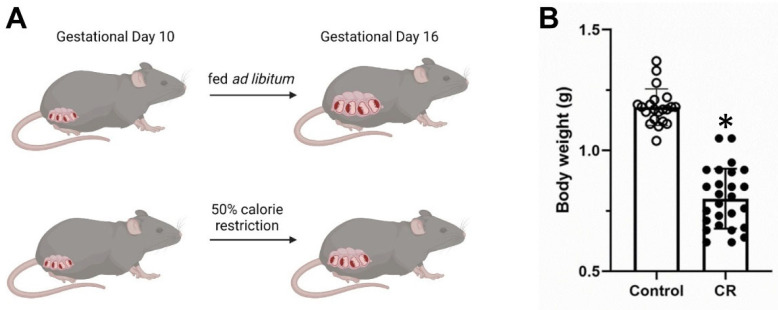
(**A**). Schematic representation of the protocol used for the in utero calorie restriction in pregnant mice. The image suggests that under calorie restriction the embryonic body weight is significantly decreased. This is verified in our experiments, (**B**) where we can see a significant decrease in the body weight of the WT embryos at gestational day 16 among the population that has been exposed to maternal calorie restriction (CR). Control: mothers fed ad libitum. * *p* < 0.05. Figure 1A was created with Biorender.com.

To ensure calorie restriction conditions in pregnant mothers that would result in low weight but viable pups, pilot experiments were performed with different time intervals of calorie restriction, as in the existing literature, but using a different strain of mice as a basis [33]. Our experience showed that if calorie restriction started from the beginning of pregnancy, no viable pups were finally born, or there were spontaneous abortions. Similarly, when the calorie restriction was extended up to birth, this also increased the spontaneous abortions and the number of non-viable pups. The protocol that was finally followed is described below and is depicted briefly in Figure 1. A male and a female mouse were housed together and every 12 h the female was checked for a vaginal plug. Once a vaginal plug was detected, the male mouse was removed from the cage and the next day was considered as day 1 of the pregnancy. From gestational day 10 up to 16, 50% of the necessary mouse diet (based on measurements of food consumption performed during the first week of pregnancy) was provided to pregnant females. Embryos were extracted under general anesthesia with isoflurane at the end of gestational day 16 and mothers were euthanized. In parallel, cages with pregnant females were maintained with ad libitum access to food for control purposes. The experiment was performed using Nrf2 heterozygous breeding pairs in order to have embryos that could potentially be WT, Nrf2KO and heterozygous.

### 2.3. RNA Preparation and Quantitative Real-Time PCR

Liver was excised from embryos and was directly immersed in RNAlater solution (ThermoFisher Scientific, Pittsburgh, PA, USA) and preserved overnight at 4 °C and at −20 °C for longer-term storage. A part of the embryonic body was preserved for gender determination by PCR and only male embryos were used. The genetic sex of the embryos was determined by PCR as previously shown using the following primers SX_F, 5ʹ-GATGATTTGAGTGGAAATGTGAGGTA-3ʹ; SX_R, 5ʹ-CTTATGTTTATAGGCATGCACCATGTA-3ʹ [34]. 10 WT and 10 Nrf2KO male embryos were used for the analyses and were selected from at least 7 different litters. RNA extraction, quantification, cDNA preparation and qRT-PCR were performed as described previously [32]. RNA purity was assessed by the absorbance ratios of 260:280 and 260:230 nm. RNA integrity was evaluated by agarose gel electrophoresis. PCR efficiency was calculated by standard curves, and the fold changes were calculated using the Pfaffl method [35]. Relative quantities were normalized to reference gene *Rps29* which was selected among 5 potential candidate reference genes (*Rps29*, *Rpl4*, *B2m*, *ActB* and *Tbp*) by employing the geNorm algorithm [36]. The primer sequences are mostly derived from Primer Bank [37] and are shown in Table 1. The selection of genes for qRT-PCR analysis was targeted. Specifically, *Nqo1* was used as the prototypical Nrf2 target gene [38] and Gsta1 and Gsta4 were selected not only because they are at least partially Nrf2-regulated [39] but they have been seen to be expressed in human embryos early in the intrauterine life [40]. The expression of genes of the integrated stress response (*Atf4* and its targets *Chac1* and *Ddit3* was measured as an index of the response to energy/nutrient deprivation. Last, as fasting is known to induce gluconeogenesis and repress lipogenesis in adult mice, key genes of each process were assessed in the calorie-restricted embryos.

### 2.4. OS Assessment

A separate experiment was performed in order to generate embryos to be used for OS assessment. The OS of embryonic liver was evaluated (a) directly by measuring superoxide radical (O_2_^•−^) levels, and (b) indirectly by measuring two specific OS markers for peroxidized lipid-induced total protein oxidative modifications: (1) malondialdehyde (MDA) bound to total proteins (Pr), designated PrMDA; (2) protein bound thiobarbituric (TBA) reactive substances (aldehydic lipid hydrocarbon fragments), designated PrTBARS. These are briefly described as follows:

O_2_^•−^ determination: Pregnant mice at the end of gestational day 16 were injected with hydroethidine (HE to final 100 μM, assuming 1 gr mouse weight corresponds to 1 mL), and after 30 min incubation they were sacrificed. Mouse mothers and their embryos were sampled for their livers which were homogenized and further processed for O_2_^•−^ determination, by a protocol by our lab described elsewhere [41]. PrMDA and PrTBARS quantification: Liver/embryo total proteins were isolated during O_2_^•−^ determination protocol execution, solubilized in ~50 µL 0.05 M NaOH, ~5 μL of which was ~20×-diluted with ddH2O and its protein content was quantified (as bovine serum albumin, BSA, equivalents) by an ultra-sensitive sensitive assay also developed by our lab [42,43]. OS marker values are expressed per mg BSA equivalents.

Total protein quantification for OS marker value expression: The total liver-proteins are isolated during execution of the aforementioned O2^•−^ assessment protocol, solubilized in ~50 µL 0.05 M NaOH, 5 μL from which is ~20×-diluted with ddH2O, and its protein content (in terms of BSA equivalents) is quantified by a sensitive protein quantification assay described elsewhere [44]. OS marker values are expressed per mg BSA equivalents.

### 2.5. Statistics

Data are expressed as means ± SD (standard deviation). The Mann-Whitney test was used for the gene expression analysis while Student’s *t*-test was employed for the other comparisons. GraphPad Prism 9 (GraphPad Software, La Jolla, CA, USA) was used for the statistical analysis and for the generation of graphs. *p* < 0.05 was considered significant.

## 3. Results

### 3.1. Maternal Calorie Restriction Induces an Antioxidant/Cytoprotective Transcriptional Response in Fetal Liver

**Figure 2:**
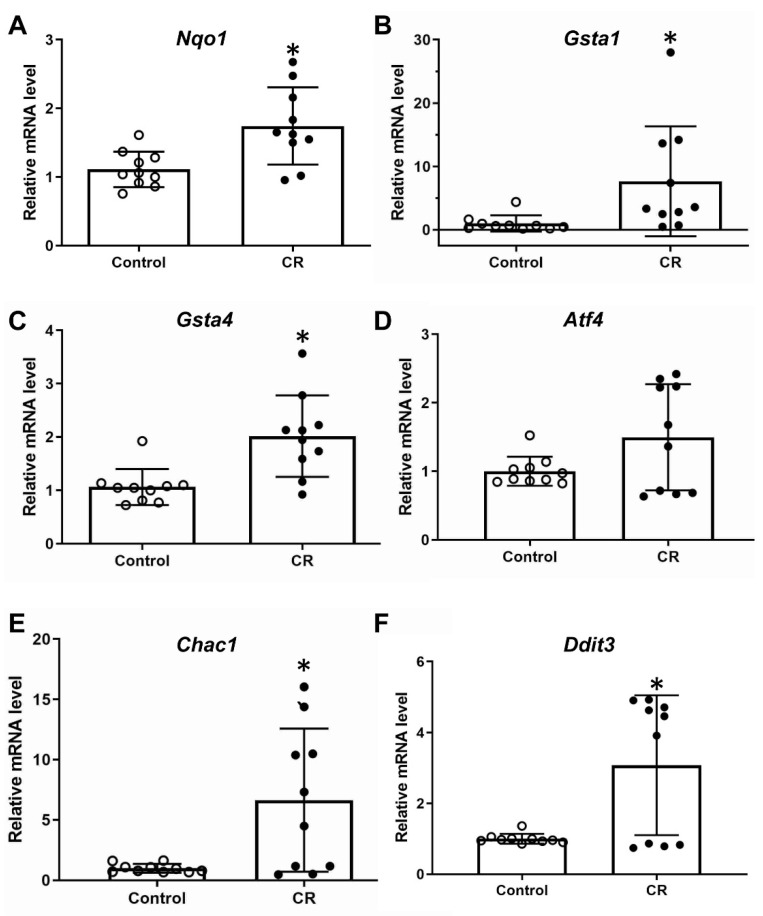
Relative mRNA expression of cytoprotective, antioxidant and stress-response genes in embryonic liver from wild-type mice: Nqo1 (**A**), Gsta1 (**B**), Gsta4 (**C**), Atf4 (**D**), Chac1 (**E**) and Ddit3 (**F**). n = 10 per group, * *p* < 0.05. Control: fed ad libitum; CR: calorie restriction.

The mRNA expression of the prototypical Nrf2 target gene Nqo1 was induced in the liver of embryos with maternal calorie restriction almost two-fold (Figure 2B). The expression of two glutathione transferases, Gsta1 and Gsta4, was also induced almost eight-fold and two-fold, respectively (Figure 2B,C). Gsts are important enzymes for detoxifying xenobiotics and secondary metabolites during OS [45] and appear to be at least partially regulated by Nrf2 [39]. The expression of some representative components of the integrated stress response (ISR) pathway, namely Atf4 and its targets Chac1 and Ddit3 [46], were examined for control purposes, as they are known, at least in adult subjects and cell lines, to be induced upon nutrient deprivation [47]. Indeed, Chac1 and Ddit3 showed a robust induction (approximately seven-fold and three-fold respectively) (Figure 2E,F) in livers of embryos with calorie restriction while Atf4 showed a trend for increase (Figure 2D).

### 3.2. Calorie Restriction Effects on Gluconeogenic and Lipogenic Gene Expression in Fetal Liver

**Figure 3:**
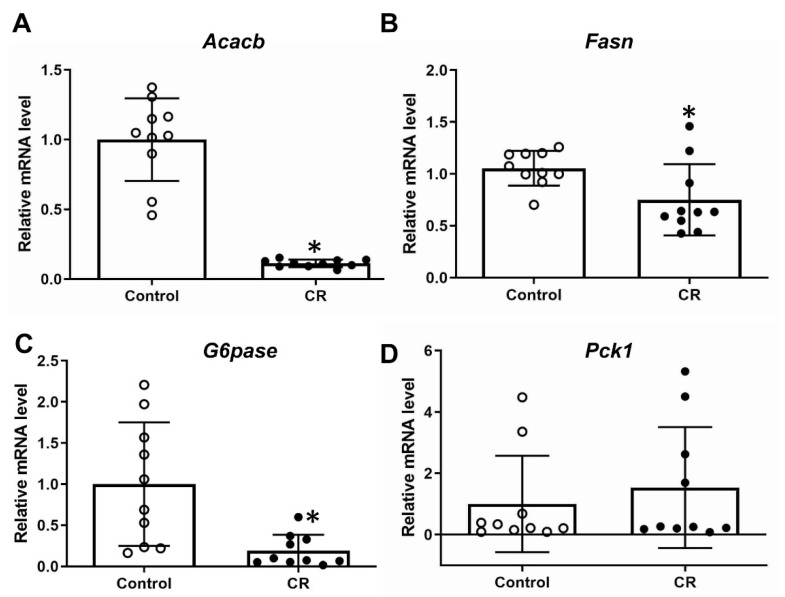
Relative mRNA expression of key lipogenic (Acacb: (**A**); Fasn: (**B**)) and gluconeogenic (G6pase: (**C**); Pck1: (**D**)) gene expression in WT embryos. n = 10 per group, * *p* < 0.05. Control: fed ad libitum; CR: calorie restriction.

The expression of two representative genes of de novo lipogenesis (Acacb and Fasn) and gluconeogenesis (G6pase and Pck1) were evaluated in response to maternal calorie restriction. Repression of both lipogenic (Figure 3A,B) and of one gluconeogenesis gene’s (G6pase) (Figure 3C) expression was observed, while no change was seen in the expression of Pckl1 (Figure 3D).

### 3.3. No Remarkable Difference in OS Was Found: Neither in the Livers of Embryos, Nor in the Livers of Their Mothers That had been Exposed to Calorie Restriction

**Figure 4:**
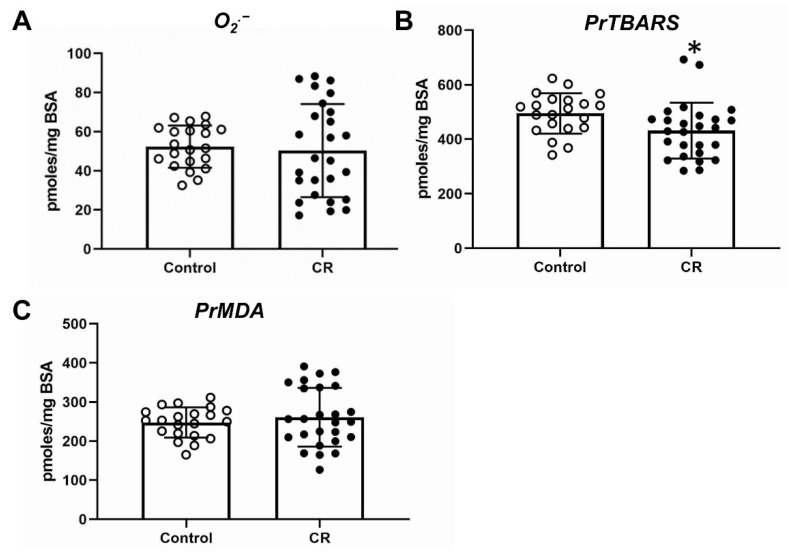
OS assessment in wild-type embryonic livers: (**A**) superoxide radical (O_2_^•−^); (**B**) PrTBARS (protein bound thiobarbituric (TBA) reactive (lipoaldehydic) substances; (**C**) PrMDA (malondialdehyde (MDA) bound to total proteins). * *p* < 0.05. Control: fed ad libitum; CR: calorie restriction.

The levels of O_2_^•−^ were assessed as a direct measure of OS. The livers of embryos did not show any difference in the superoxide radical levels, without or with maternal calorie restriction (Figure 4A). The levels of PrTBARS, an indirect measure of oxidative stress, were found roughly 13% decreased in the livers of calorie-restricted embryos (Figure 4B). This difference is statistically significant but with a physiological significance that remains to be evaluated. Another indirect marker of OS PrMDA showed no difference between the two groups of murine embryos (Figure 4C).

**Figure 5:**
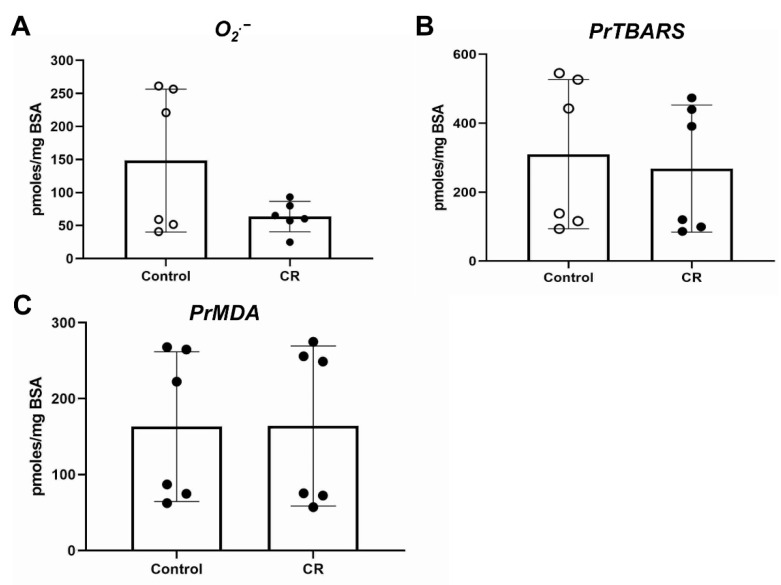
OS assessment in livers of WT mothers of embryos shown in Figure 4: (**A**) superoxide radical (O2^•−^); (**B**) PrTBARS (protein bound thiobarbituric (TBA) reactive (lipoaldehydic) substances; (**C**) PrMDA (malondialde-hyde (MDA) bound to total proteins). Control: fed ad libitum; CR: calorie restriction.

No significant difference was found in either O2^•−^, PrTBARS or PrMDA in the livers of the WT mothers (Figure 5).

### 3.4. Lack of Nrf2 Partially Attenuates the Antioxidant Transcriptional Response to Maternal Calorie Restriction in Murine Fetal Livers

**Figure 6:**
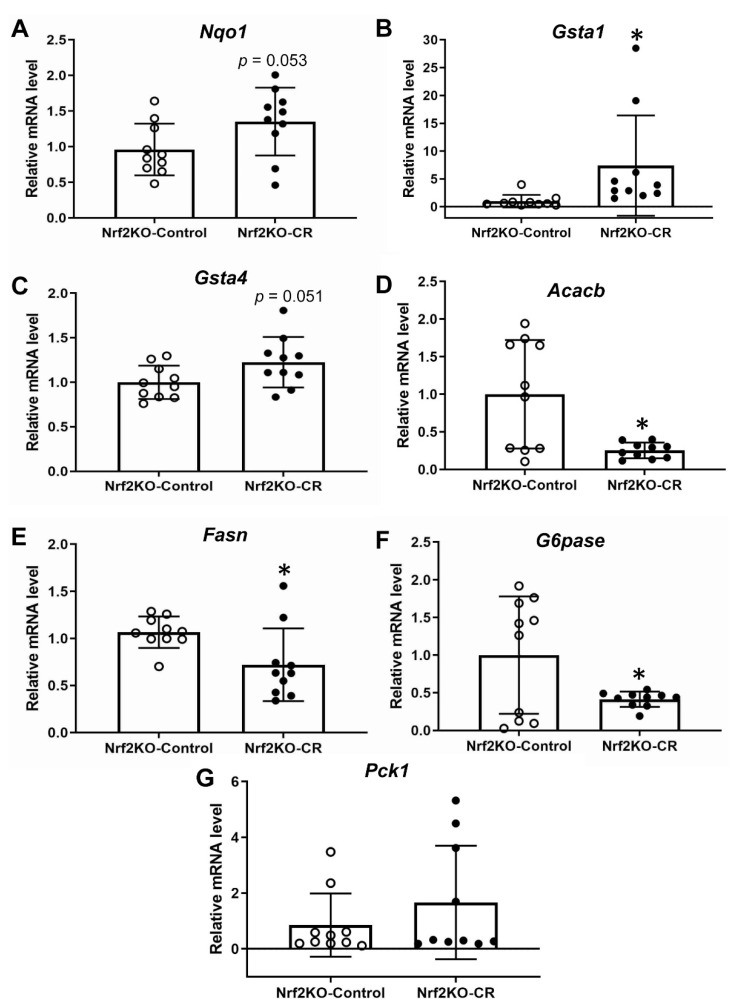
Relative mRNA expression of cytoprotective/antioxidant (Nqo1 (**A**), Gsta1 (**B**), and Gsta4 (**C**)); key lipogenic (Acacb (**D**), Fasn (**E**)); and gluconeogenic enzymes (G6pase (**F**); Pck1 (**G**)) in Nrf2 knockout embryos. n = 10 per group, * *p* < 0.05. Nrf2KO-Control: Nrf2 knockout embryos fed ad libitum; Nrf2KO-CR: calorie-restricted Nrf2 knockout embryos.

When the same experiment with maternal calorie restriction was repeated using Nrf2 knockout mice, Nqo1 (Figure 6A) and Gsta4 (Figure 6C) gene expression was not significantly induced after calorie restriction, indicating an Nrf2-dependent effect. The induction of Gsta1 was maintained even in the absence of Nrf2 (Figure 6B) suggesting that in this context Gsta1 is mostly Nrf2-independent. The repression of Acacb, Fasn and G6pase (Figure 6D–F) is also maintained in the absence of Nrf2 with a few differences in the degree of repression. Specifically, calorie restriction led to almost nine-fold and five-fold repression of Acacb and G6pase expression, respectively, in WT mice (Figure 3A) and to nearly four-fold and two-and-a-half-fold respectively in the Nrf2KO mice (Figure 6D). No change was found in the Pck1 gene expression in the absence of Nrf2 (Figure 6G), as was also the case in WT mice (Figure 3D).

## 4. Discussion

Herein, to the best of our knowledge, we showed for the first time that maternal calorie restriction by 50% induced the transcription of antioxidant and cytoprotective genes in fetal murine liver in an at least partially Nrf2-independent manner. In this context, the induction of the prototypical Nrf2 target gene *Nqo1* suggests that Nrf2 signaling pathway was activated. It appears that this induction of cytoprotective and antioxidant genes is not secondary to an increased OS, as direct and indirect indexes of ROS indicate that there is mostly no remarkable change in ROS levels. Thus, other mechanisms of Nrf2 pathway activation in embryos during maternal calorie restriction should be considered. For instance, βTrcp and GSK-3 could theoretically play a role [48], as they can affect Nrf2 pathway activity independently of Keap1. Moreover, other natural metabolites such as itaconate could interact with Keap1 independently of ROS and activate Nrf2 signaling [49].

By using a well-established model of intrauterine growth retardation due to maternal calorie restriction by 50% [33] between gestational days 10 to 16, we mimicked to some degree the effects of famine in human mothers that resulted in low-birth-weight offspring. Instead of studying the effects of this in utero calorie restriction during adult life in the offspring, we focused our study on the acute effects of calorie restriction in embryonic livers so as to better understand the intrauterine environment that this metabolic stress creates. A previous study using rat mothers who were fasted for 48 h, from gestation day 19 to 20 [50], also showed that this resulted in smaller size embryos. The present model of intrauterine growth restriction could also be compared with other relevant models, related or not to metabolism, such as fasting of the mothers or ligation of uterine arteries in order to examine if this transcriptional response is unique to the model used herein.

The use of direct O_2_^•−^ and indirect (PrTBARS, PrMDA) indices (Figure 4) in order to evaluate the OS in the murine liver provides a methodological advantage in our approach, as it is innovative from two different perspectives. Firstly, the in vivo measurement of O_2_^•−^, which is the main initiator of OS-induced ROS generation, is done for the first time in whole animal tissue by an ultrasensitive methodology originally developed by our lab for that particular approach. Secondly, the selection of the OS indices such as PrTBARS and PrMDA is innovative and informative, as they are a measure of the destructive oxidative effect of ROS (e.g., O2^•−^) on proteins, the major organic molecular fraction of every life form, and because they are very sensitive indicators of early OS, being the early products of the ROS-induced chain-like propagated peroxidation of lipids, another major class of biological molecules.

The intrauterine calorie restriction in our study did not result in changes in the ROS levels, which were mostly similar between the control and the calorie-restricted groups. A slight decrease in PrTBARS levels after calorie restriction indicates that the overall trend is that the ROS levels remained mostly the same, and possibly with a tendency to decrease in embryos of calorie-restricted mothers (Figure 4). Similarly, no difference in ROS was detected in the livers of the mothers after calorie restriction (Figure 5) with high variability in the measurements. An interesting observation is that the absolute levels of O_2_^•−^ are higher in the mothers rather than in the embryos, while the levels of PrTBARS and PrMDA are mostly within the same range between mothers and embryos. This could mean that even though the embryos have lower levels of O_2_^•−^, a possibly “immature” antioxidant cytoprotective system leads to similar destructive effects on lipids and proteins, as indicated by the levels of PrTBARS and PrMDA.

Calorie restriction in adult life is generally known to increase longevity and one of the proposed mechanisms is the reduction of ROS (reviewed in [51]). The lowering of the ROS level is usually attributed to the decreased ROS generation by mitochondria due to the limited availability of “fuel”. However, some reports show an increased expression of antioxidant enzymes [30,52] dependent on and independent of Nrf2 after calorie restriction that could contribute to the decrease of ROS levels. Even though it is not ideal to extrapolate findings from adult mice to embryos, this induction of Nrf2-dependent and independent antioxidant enzymes is also found in our study (Figure 2). It is hard to explain the physiological significance of this finding in utero and more research is warranted in order to follow up with these mice in adult life to check if these changes in antioxidant/cytoprotective gene expression are sustained, and also if it has a functional significance. For instance, mice that were exposed to calorie restriction in utero can be compared with control mice in terms of their response to a well-established toxic insult such as treatment with acetaminophen [53]. Mice with loss of Nrf2 function are more sensitive to acetaminophen toxicity [54] while mice with an activated Nrf2 pathway are more resistant to it [55].

As no changes are detected in oxidative stress markers in the embryos under calorie restriction, the activation of the Nrf2 pathway is not due to increased ROS levels that interact with Keap1 cysteines. Hence, the mechanism of Nrf2 pathway activation in this context requires further investigation. For instance, this mechanism might be Keap1-independent and, for example, include the involvement of the Glycogen Synthase Kinase 3/β-TrCP Axis [56]. Calorie restriction is known to lead to reduced GSK3/β levels [57], which could reduce the TrCP-mediated degradation of Nrf2 and thus enhance Nrf2 signaling. Of course, the possibility that other electrophiles rather than ROS could modify the sulfhydryl groups of Keap1 and activate Nrf2 pathway could not be excluded.

Another interesting finding of our study is the upregulation of components of integrated stress response (ISR), and notably Atf4 target genes Chac1 and Ddit3 (Figure 2E,F). It is known that ISR is activated from several cell stresses including nutrient deprivation [46]. The most prominent gene upregulated via this mechanism is the transcription factor Atf4, which is considered an important regulator of nutrient sensing and protein turnover [58,59]. Pathologies associated with dysregulated ISR include cognitive and neurodegenerative disorders, diabetes mellitus and cancer [46]. Although the expression of Atf4 targets Chac1 and Ddit3 was mostly used to demonstrate that the embryos are “sensing” the calorie and nutrient deprivation, it also provides evidence that the ISR system is active in utero and responds to calorie restriction, given that ISR has not been studied extensively in embryos. In our mouse model, which is based on calorie deprivation by restricting access to food, it is impossible to distinguish whether the effects seen are due to calorie restriction per se or due to the lack of specific nutrients such as proteins, vitamins etc. The ISR is known to be implicated in the pathogenesis of preeclampsia [60] and in neurodevelopmental deficits [61,62], to name a few of prenatal pathologies. Given that there are some indications that Nrf2 can possibly co-operate with Atf4 signaling in order to induce an antioxidant-cytoprotective response [63], it would be plausible that such an interaction may also be present in life in utero and consist one of the mechanisms of embryonic protection. Furthermore, one of the results of ISR is the decrease in global protein synthesis [64]. This is something we have not evaluated in our model, but it was found true in an alternative rat model of maternal fasting [50]. It would also be interesting to see if the ISR and the Nrf2 antioxidant response are induced in embryonic liver by alternative, more targeted, methods of nutrient deprivation such as amino acid deprivation, and examine the resulting phenotypic differences.

In the present study we also examined the effects of maternal calorie restriction on key gluconeogenic and lipogenic enzymes in fetal liver. Our findings support the proposition that maternal calorie restriction reduces lipogenesis in embryonic liver and represses the m RNA expression of G6pase (Figure 3). Lipogenesis and gluconeogenesis are important in the metabolic response of mice to energy/substrates deprivation. It is also worth mentioning that, as described in the introduction, Nrf2 has been described to repress these pathways under conditions of obesity and insulin resistance [28,65]. It is true that in embryos that rely on supply of maternal nutrients, the regulation of these processes is more complicated. Under normal conditions embryos do not have to perform gluconeogenesis but under abnormal conditions such as intrauterine growth retardation, increased gluconeogenesis is required in order to provide glucose to the embryo [66]. Hence, it is hard to explain the physiological significance of the repressed G6pase (Figure 3C) in embryonic liver seen after maternal calorie restriction, while the levels of the other key gluconeogenic enzyme Pck1 remain unchanged (Figure 3D). Moreover, the transcriptional repression of lipogenic enzymes *Acacb* and *Fasn* in the livers of embryos under calorie restriction theoretically could make sense given that under conditions of energy deprivation anabolic processes such as lipogenesis are repressed. This repression of G6pase and the two lipogenic gene expression was also present in the absence of Nrf2 (Figure 3A–C, Figure 6D–F) but to a slightly lesser degree for G6pase and Acacb, which could be attributed to the known repressive effect of Nrf2 signaling in these genes. Specifically, G6pase gene expression was repressed five-fold in WT embryos and two-and-a-half-fold in Nrf2KO after calorie restriction; Acacb was repressed nine-fold in WT versus four-fold in Nrf2KO. This repression was also detected in the embryonic liver microarray data of a previous study with fasting rat mothers (n = 3, data not verified by qPCR, GEO accession GSE77112 using GEO2R [50]).

In this previous study using fasting rat mothers, and employing a microarray analysis of embryonic liver (GEO accession GSE77112 using GEO2R [50]), no statistically significant difference can be found in Nqo1, Gsta1 and Gsta4 (n = 3, data not verified by qPCR). Last, in this study, maternal calorie restriction led to induction of a transcriptional antioxidant/cytoprotection program in fetal liver in an at least partially Nrf2-dependent manner (Figure 2 and Figure 6). Some limitations of our study have to be acknowledged. First, liver is the only embryonic tissue studied for practical purposes (easily visible and excisable without contamination from neighboring tissues). It would be also interesting to see if this antioxidant response and the ROS levels are similar in other embryonic structures and the placenta itself, given that placenta shows mitochondrial aberrations due to calorie restriction [67]. Furthermore, the inclusion of protein expression data or enzyme activities would ideally be a nice addition to this study but given the limitation of the tissue size our analyses were limited to qPCR-based mRNA gene expression. Since our study was focused on a limited set of genes, a more unbiased approach using RNA-Seq based transcriptomics analysis could aid us to evaluate the presented pathways globally and potentially detecting new enriched pathways that are worth of further studies. Finally, a targeted metabolomics/lipidomics approach in the embryonic liver would give insights on the functional significance of these observed transcriptomic changes in gluconeogenic and lipogenic enzymes.

## 5. Conclusions

In conclusion, we showed that maternal calorie restriction induced transcriptionally cytoprotective and antioxidant enzymes in the fetal liver along with genes related to integrated stress-response. These results warrant further investigation in terms of Nrf2 dependency, as we described here an at least partially Nrf2-dependent mechanism. Last, the effects of the observed changes in utero remain to be studied in adult life. Specifically, the observed changes of antioxidant/cytoprotective genes as well as metabolic genes could have consequences during the adult life of mice related to their responses to nutrient deprivation or excess, and to toxic stimuli.

## Figures and Tables

**Table 1:** Primers sequences used for qRT-PCR.

Gene (NCBI ID)	Forward Primer	Reverse Primer
*Acacb (100705)*	CCTTTGGCAACAAGCAAGGTA	AGTCGTACACATAGGTGGTCC
*Atf4 (11911)*	CCTGAACAGCGAAGTGTTGG	TGGAGAACCCATGAGGTTTCAA
*Chac1 (69065)*	CTGTGGATTTTCGGGTACGG	CCCCTATGGAAGGTGTCTCC
*Ddit3 (13198)*	AAGCCTGGTATGAGGATCTGC	TTCCTGGGGATGAGATATAGGTG
*Fasn (14104)*	GGAGGTGGTGATAGCCGGTAT	TGGGTAATCCATAGAGCCCAG
*Gsta1 (14857)*	AAGCCCGTGCTTCACTACTTC	GGGCACTTGGTCAAACATCAAA
*Gsta4 (14860)*	TGATTGCCGTGGCTCCATTTA	CAACGAGAAAAGCCTCTCCGT
*G6pase (14377)*	CTAGCTTTGATCTGGTTGTCAG	GTTGAACCAGTCTCCGACCA
*Nqo1 (18104)*	CATTCTGAAAGGCTGGTTTGA	CTAGCTTTGATCTGGTTGTCAG
*Pck1 (18534)*	CTGCATAACGGTCTGGACTTC	CAGCAACTGCCCGTACTCC
*Rps29 (20090)*	TCTACTGGAGTCACCCACGGAA	GGAAGCACTGGCGGCACA

## Data Availability

All of the data is contained in the article. Raw data supporting the results of this study are available upon request to the corresponding author.
